# Different tissue reaction of oesophagus and diaphragm after mesh hiatoplasty. Results of an animal study

**DOI:** 10.1186/1471-2482-8-7

**Published:** 2008-04-12

**Authors:** Jens Otto, Daniel Kämmer, Petra Lynen Jansen, Michael Anurov, Svetlana Titkova, Alexander Öttinger, Raphael Rosch, Volker Schumpelick, Marc Jansen

**Affiliations:** 1 Department of Surgery, University Clinic RWTH Aachen, Pauwelsstrasse 30, 52057 Aachen, Germany; 2 Department of Physiology, Russian State Medical University, Ostrovityanova 1, Moscow 117347, Russia

## Abstract

**Background:**

Laparoscopic mesh-reinforcement of the hiatal region in the treatment of gastroesophageal reflux disease (GERD) and paraesophageal hernia (PEH) reduces the risk of recurrence. However, there are still controversies about the technique of mesh placement, shape, structure and material. We therefore compared tissue integration and scar formation after implantation of two different polypropylene-meshes in a rabbit model.

**Methods:**

A total of 20 female chinchilla rabbits were included in this study. Two different meshes (Polypropylene PP, Polyglecaprone 25 Composite PP-PG) were implanted on the abdominal diaphragm around the oesophagus. After 3 months the implanted meshes were excised en-bloc. Histological and morphological analyses were carried out accordingly proliferation rate, apoptosis and collagen type I/III ratio.

**Results:**

Regarding proliferation rate of oesophagus PP (9.31 ± 3.4%) and PP-PG (13.26 ± 2.54%) differ in a significant (p = 0.0097) way. In the diaphragm we found a significant (p = 0.00066) difference between PP (9.43 ± 1.45%) and PP-PG (18.73 ± 5.92%) respectively. Comparing oesophagus and diaphragm we could prove a significant difference within PP-PG-group (p = 0.0195). Within PP-group the difference reached no statistical significance (p = 0.88). We found analogous results regarding apoptosis.

Furthermore, there is a significant (p = 0.00013) difference of collagen type I/III ratio in PP-PG (12.28 ± 0.8) compared to PP (8.44 ± 1,63) in case of oesophageal tissue. Concerning diaphragm we found a significant difference (p = 0.000099) between PP-PG (8.85 ± 0.81) and PP (6.32 ± 1.07) as well.

**Conclusion:**

The histologic and morphologic characteristics after prosthetic enforcement of the hiatus in this animal model show a more distinct tissue integration using PP-PG compared to PP. Additionally, different wound healing and remodelling capability influence tissue integration of the mesh in diaphragm and oesophagus.

## Background

Laparoscopic repair of gastroesophageal reflux disease (GERD) and paraesophageal hernias (PEH) has become the treatment of choice [1]. Although there is an increasing experience with laparoscopic paraesophageal hernia (LPEH) repair, studies observed recurrence rates of up to 43% with simple, primary suture of the hiatus [2]. Furthermore, Granderath et al. noticed a high rate of intrathoracic wrap migration of 26% in patients undergoing laparoscopic fundoplication (LF) with primary sutured hiatal repair [3]. Kamolz et al. observed that mesh-reinforcement of the hiatal crura reduced the risk of recurrent hiatal hernia and led to an improved quality of life compaired to patients without mesh prothesis [4]. It seems as if use of prosthetic inforcement of the hiatus becomes routine in clinical practice [5-7]. However, authors continue reporting of stricture, dysphagia, ulceration, perforation or even mesh migration into the oesophagus caused by use of alloplastic mesh material for hiatoplasty [7-9]. Moreover, there are still controversies about the technique of mesh placement, shape, structure and material [7,10,11]. Various materials have been investigated (polypropylene mesh, polytetrafluoroethylene mesh, acellular dermal allograft). The results of these studies base on clinical outcomes in most cases [1,5,12-14]. Desai et al. presented a canine model and reported on histological results one year after bioprosthetic repair of paraoesophageal hernia with a new small-intestinal submucosa mesh (SIS). They found no evidence of erosion of SIS mesh into the eosophagus [15]. Following this canine model, we performed an animal study to examine functional and histological changes in the distal eosophagus after implantation of two different mesh material [polypropylene (PP), Prolene^®^; polypropylene-polyglecaprone 25 composite (PP-PG), Ultrapro^®^]. Data of the clinical outcome of this study were already published by our group [16]. We observed distinctive mesh shrinkage after three months in all animals. Some meshes had lost up to 50% of their original size. We found a delayed passage of fluid into the stomach of all operated animals. Furthermore we found a mesh migration into the esophageal wall in six out of seven animals (PP) and five out of nine animals (PP-PG), respectively [16].

In the present study we assessed the histologic characteristics, tissue integration and scar formation after prosthetic enforcement (PP and PP-PG) of the hiatus in a rabbit model.

## Methods

### Animals and Anaesthesia

A total of 20 female chinchilla rabbits (mean body weight 2.5 kg +/- 0.3 kg) were included in this study, which was performed according to the rules of the "Deutsche Tierschutzgesetz", to the NIH guidelines for the use of laboratory animals and to the GLP standard (good laboratory practice, ISO 10993-6). The animals were kept in single cages under standard laboratory conditions with balanced pellet diet and water ad libitum. Rabbits were randomly assigned to two different groups of equal numbers and the surgical procedures were performed under sterile conditions and general anaesthesia by intravenous administration of ketamine (Ketamin 10%, Sanofi-Ceva, Dusseldorf, Germany) and Xylazine (Rompun 2%. Bayer, Leverkusen, Germany) [17]. After hair removal, the abdomen was opened by an upper midline incision. The stomach and the distal oesophagus were exposed. Two different meshes (Polypropylene (PP), Prolene^® ^– Polyglecaprone 25 Composite (PP-PG), Ultrapro^® ^Table 1) were implanted on the abdominal diaphragm around the oesophagus with a circular distance of 3 mm. The meshes had a diameter of 2 cm and were fixed to the diaphragm with 4 Polypropylene (6-0) single stitches. Finally the abdominal cavity was closed by two running sutures of 3-0 polyglycolic acid. After 3 months the animals were sacrificed by a letal dose of pentobarbital sodium (Narcoren, Rhone Merieux, Laupheim, Germany). The abdominal cavity was reopened via a u-shaped incision in the upper abdomen for complete exploration. The distal oesophagus and the diaphragm including the implanted mesh were excised entirely.

**Table 1:** Textile properties of the investigated mesh materials

	Prolene^® ^(PP)	Ultrapro^® ^(PP-PG)
Polymer	Non-absorbable Polypropylene	Composite of absorbable Polyglecaprone and non-absorbable Polypropylene
Structure	Mono-filament	Mono-filament
Weight (g/m^2^)	108,5	55 (28 after resorption)
Pore diameter (mm)	1,6	3

### Histological analysis

Tissue specimens were fixed in 10% formaldehyde and embedded in paraffin. The tissue was dissected into two parts, containing mesh and diaphragm on the one hand and mesh and oesophagus on the other hand. Histological examination was performed on 3 mm sections after haematoxylin and eosin staining (H&E). Percentages of proliferating and apoptotic cells at the interface of mesh to host tissue were investigated following immunohistochemical staining as described previously [18]. Monoclonal mouse anti-rat Ki67 (MIB5) for cell proliferation rate (1:10, Dako, Glostrup, Denmark) as well as monoclonal mouse anti-rat (ED 1) for selective staining of macrophages (1:250, DPC Biermann, Bad Nauheim, Germany) were used. TUNEL histochemistry for apoptosis and DNA strand breaks were performed by in situ apoptosis detection kit (ApopTag Peroxidase Kit, S7100, Intergen, Oxford, UK). Percentage of positively stained cells was assessed within the interface of meshes to host tissue (× 400, area 100 × 100 μm directly next to the mesh fibres) using a digital image-analyzing software (Image-Pro Plus; Media Cybernetics, Silver Spring, Md., USA). For each mesh and implantation period 15 measurements were performed.

### Cross-Polarization Microscopy

For cross-polymerization microscopy, 5-μm sections of the centre of the mesh samples and of the surrounding perifilamentary tissue were stained for 1 h in Picrosirius solution (0.1% solution of Sirius Red F3BA in saturated aqueous picric acid, pH 2) according to the method of Junqueira et al. [19]. The sections were then washed for 2 min in 0.01 *N *HCl, dehydrated, cleared, and mounted in synthetic resin. To analyze the collagen type I/III ratio, tissue samples were investigated using cross-polarization microscopy. Thicker collagen type I fibres were stained in red-orange shades, whereas thinner collagen type III appeared as pale-green shades. For each sample, standard regions within the interface (× 400, area 100 × 100 μm directly next to the mesh fibres) were captured by a digital camera (C-3030; Olympus, Hamburg, Germany). The amount of collagen type I and type III was obtained using digital image-analyzing software (Image-Pro Plus; Media Cybernetics, Silver Spring, Md., USA). Results are expressed as quotient of collagen type I to III. For each mesh and implantation period, 30 measurements were performed.

### Statistics

Statistical analysis was carried out using Statistical Package for Social Sciences (SPSS^®^)-software. Data were organized according to mesh modification and site of implantation ("oesophagus" and "diaphragm"). The relative amount of proliferating and apoptotic cells were tested for normal distribution by the Kolmogoroff-Smirnov test. Statistical analysis was performed by a two-way ANOVA with pairwise comparison. P-values of 0.05 were considered to be significant. All data are presented as means ± standard deviation.

## Results

Overall, macroscopic clinical observation after the initial surgical procedure was uneventful in all animals. Four animals (3: PP; 1: PP-PG) died due to pneumonia. In an adjacent necropsy we found no complications caused by the surgical procedure. The remaining 16 rabbits were included into the study after the complete observation period of three months.

**Figure 1:**
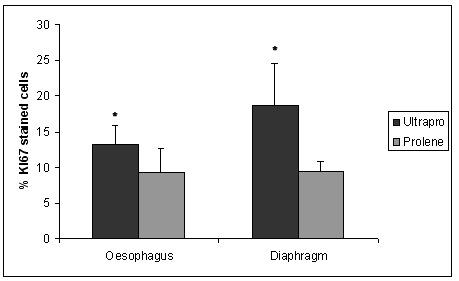
**Proliferation rate**. (percentage of KI67 stained cells; * = statistically significant).

**Figure 2:**
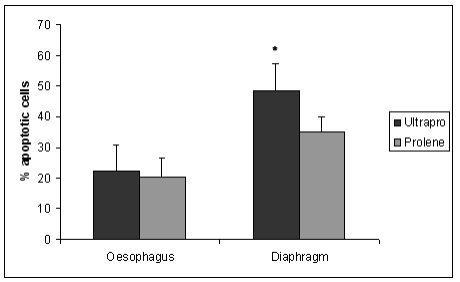
**Apoptosis rate**. (percentage of TUNEL stained cells; * = statistically significant).

**Figure 3:**
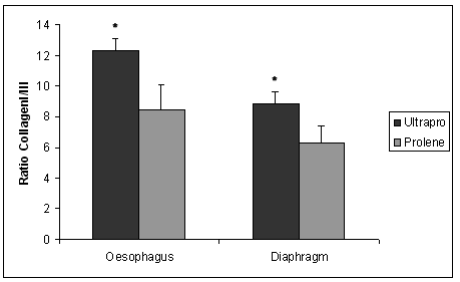
**Ratio Collagen I/III**. (* = statistically significant).

We found an increased proliferation rate (Figure 1), apoptosis rate (Figure 2) and collagen type I/III ratio (Figure 3) in PP-PG-group compared to PP-group in both oesophagus and diaphragm. Comparing oesophagus and diaphragm tissue reaction we could show an increased proliferation rate and apoptosis rate and a reduced collagen type I/III ratio. Evaluating the individual differences we explored a statistical significance in all apart from two cases. The definite results and P-values of differences are presented in Table 2.

**Table 2:** Results (values ± SD; P-values)

	**Oesophagus**			**Diaphragm**			**Oesophagus vs. Diaphragm**	
**Mesh-material**	**PP**	**PP-PG**	**Significance of difference**	**PP**	**PP-PG**	**Significance of difference**	**PP**	**PP-PG**

**Proliferation rate [%]**	9.31 ± 3.4	13.26 ± 2.54	p = 0.0097 significant	9.43 ± 1.45	18.73 ± 5.92	p = 0.00066 significant	p = 0.88 *not significant*	p = 0.0195 significant
**Apoptosis rate [%]**	20.06 ± 6.42	22.25 ± 8.67	p = 0.52 *not significant*	35.07 ± 4.88	48.47 ± 8.82	p = 0.00104 significant	p = 0.000375 significant	p = 0.000027 significant
**Cross polarization microscopy [ratio]**	8.44 ± 1,63	12.28 ± 0.8	p = 0.00013 significant	6.32 ± 1.07	8.85 ± 0.81	p = 0.000099 significant	p = 0.0095 significant	p < 0.0001 significant

## Discussion

Prosthetic enforcement of the hiatal hernia during laparoscpopic hiatal hernia repair leads to a decreased recurrence rate. However, the review of several available studies show a controversy regarding different mesh materials, structures and shape for narrowing the oesophageal hiatus [20]. The evaluation of different types of hiatal meshes base largely on functional and clinical results. This study shows morphological and histological changes in an animal model using established methods originating from research investigating other types of mesh repair (e.g. incisional hernia) [21-23].

Tissue response to PP mesh and PP-PG mesh as well was characterized by a moderate inflammatory tissue reaction limited to the perifilamentory region. Investigating the cell proliferation rate (Ki 67 staining) and apoptosis rate (TUNEL staining) we found a more distinct perifilamentory mesh integration and tissue remodeling in case of PP-PG. We could even prove statistical significance between both mesh modifications, apart from TUNEL staining in the oesophagus group. Interestingly, comparing oesophageal and diaphragm part of the mesh integration we found significant differences irrespective of the used mesh material. The small and different number of animals in both groups in combination with high standard deviations have to be considered valuating the statistical results. We explored the perifilamentory tissue reaction and remodeling with regard to collagen. Collagen represents the quantitatively most abundant protein of the body and is the most important scleroprotein of the extracellulaer matrix (ECM) [24]. During transformation of initial granulation tissue into connective tissue, immature type III collagen is replaced by mature type I collagen. Intermolecular cross linkage between collagen type I and III results in mechanical stability and tensile strength. An increased ratio of collagen type I to III corresponds with an improvement of tissue stability [24]. We found an increased ratio of collagen I/III and therefore a more mature scarring in case of PP-PG compared to PP in both oesophageal and diaphragm part of the mesh. This effect may base on a better mesh incorporation due to a more effective perifilamentory tissue response in case of PP-PG. Burger et al. explored similar results in a rat model, evaluating prosthetic meshes for ventral hernia repair. Adhesion formation, mesh incorporation, tensile strength, shrinkage, mesh infection, and tissue response were scored and compared [25]. Furthermore, we could show in this study a reduced ratio of collagen I/III in case of diaphragm part of the mesh. In accordance with our explanatory model, we found a decreased mechanical stability and tensile strength in the diaphragm part of the mesh. Diaphragm movements cause changing tension direction and mechanical stress during the time of wound healing and transformation into connective tissue. This might explain the less effective mesh-tissue integration and the smaller ratio of collagen I/III in diaphragm compared to oesophagus.

**Figure 4:**
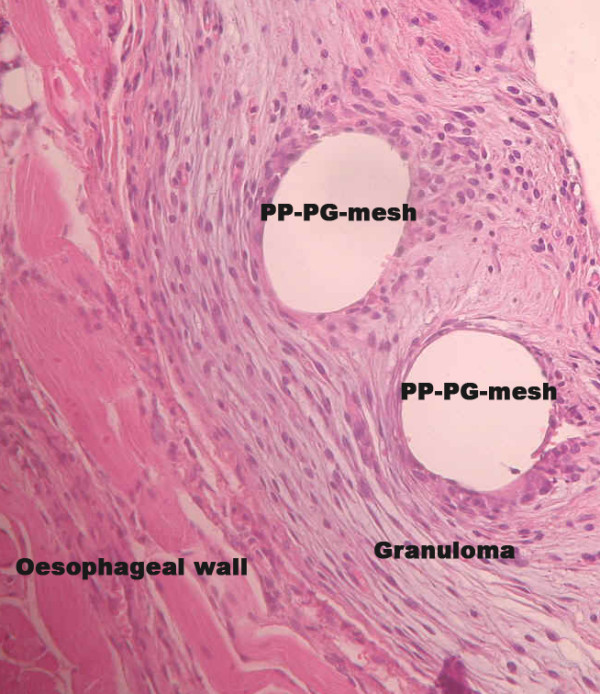
**Mesh-tissue integration of PP-PG-mesh (Ultrapro^®^) in the oesophageal part**. (H&E; ×200).

**Figure 5:**
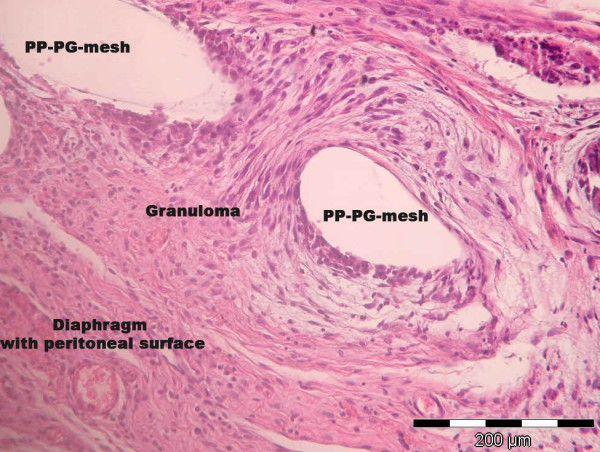
**Mesh-tissue integration of PP-PG-mesh (Ultrapro^®^) in the diaphragm**. (H&E; ×200).

Exploring the structure of perifilamentary granulomas regarding morphological aspects, we observed a loose-fitting and less structured configuration in the diaphragm part of the tissue response (Figure 4 and 5). This is in line with the described lower rate of collagen I/III in this area. Greca et al. could show a positive correlation between mesh porosity and densitometric proportion of mature (type I) collagen using different meshes for repair of abdominal wall defects in dogs [26]. This conforms to the higher rate of collagen I/III in case of PP-PG compaired to PP in the present study. The causation of proliferation, apoptosis and ratio of collagen I/III during the wound healing process refers specifically to the perifilamentory granulomas. In this context the tissue response and the mesh integration of the lightweight composite mesh (PP-PG) in the present study has to be seen, too. The histological analysis does not refer to foreign body reactions like adhesions, fibrosis or seroma [27].

One might subsume these results under two different aspects. On the one hand we could prove an overall acceptable biocompatibility for both PP and PP-PG with a better tissue integration of the lightweight composite mesh (PP-PG). On the other hand we could display the different organ-dependent wound healing and capability of mesh integration. It must be stated that animal studies have their limitations and cannot directly be transferred to humans, but this type of study of tissue reaction and mesh integration is not possible to do on humans. Nevertheless this study shows the complexity of hiatal mesh reinforcement. In contrast to mesh repair of an abdominal wall hernia we are confronted with a three dimensional moving system consisting of the diaphragm, a horizontal muscle layer with peritoneal surface and the oesophagus, a vertical hollow organ of the digestive tract. This might be one reason for complications of hiatal mesh reinforcement like stenosis and transmural migration as explored and presented in the clinical analysis of this study. We found a prolonged passage of contrast medium into the stomach in all operated animals, irrespective of the implanted mesh. In addition we could show a high migration rate into the eosophageal wall, even into submucosa in case of PP mesh [16]. Here we are in line with Hergueta-Delgado and co-workers. They could show that hiatal reinforcement with mesh is an effective procedure to reduce recurrence, but it comes at a price, namely, migration of the mesh into the esophageal lumen [28]. Both Granderath and Casaccia have investigated the influence of different mesh shape (A-shape, U-shape) for the hiatal reinforcement [5,13]. The results of the presented study show that a circular implantation of meshes around the eosophagus leads to a high rate of mesh migration [16].

Concerning the present status, the indication for mesh hiatoplasty should be carried out very carefully, but with regard to its effectiveness to reduce recurrence rate of hiatal hernia further development is needed.

## Conclusion

The histologic and morphologic characteristics after prosthetic reinforcement of the hiatus in this animal model show a slightly improved tissue integration using PP-PG compared to PP. Additionally, different wound healing and remodelling capability influence the mesh tissue integration in diaphragm and oesophagus. The clinical and histological results of both PP and PP-PG of the present study argue for a contained use for mesh hiatoplasty.

## Competing interests

The author(s) declare that they have no competing interests.

## Authors' contributions

JO, MA, ST, AÖ and MJ carried out the operation and the clinical examination of the animals. JO, DK and PLJ carried out the histological and morphological analysis. RR carried out the Cross-Polarization Microscopy. JO, VS and MJ participated in the design of the study and performed the statistical analysis. JO, DK and MJ drafted the manuscript. All authors read and approved the final manuscript.

## Pre-publication history

The pre-publication history for this paper can be accessed here:


